# Supplementary material for: Subcritical Water Extraction of Undaria pinnatifida: Comparative Study of the Chemical Properties and Biological Activities across Different Parts
Source: Mar Drugs. 2024 Jul 27;22(8):344. doi: 10.3390/md22080344 (PMC11355683; doi:10.3390/md22080344)
Supplement: Supplementary file 1 [file marinedrugs-22-00344-s001.zip › marinedrugs-3128570-supplementary.pdf]

Subcritical Water Extraction of *Undaria pinnatifida*: Comparative Study of the Chemical Properties and Biological Activities across Different Parts

Supplementary Data

3.5.2. Antihypertensive activity

Supplementary Information: Preparation of Working Solutions and Sample Solutions

Enzyme Working Solution

1. Preparation of Enzyme B Solution:
   - Add 2 mL of deionized water to the Enzyme B vial.
   - Mix thoroughly to dissolve completely.
2. Preparation of Enzyme Working Solution:
   - Add 1.5 mL of the prepared Enzyme B solution to the Enzyme A vial.
   - Mix thoroughly to prepare the Enzyme Working Solution.

Indicator Working Solution

1. Preparation of Enzyme C Solution:
   - Add 3 mL of deionized water to the Enzyme C vial.
   - Mix thoroughly to dissolve completely.
2. Preparation of Coenzyme Solution:
   - Add 3 mL of deionized water to the Coenzyme vial.
   - Mix thoroughly to dissolve completely.
3. Preparation of Indicator Working Solution:
   - Combine 2.8 mL of the Enzyme C solution with 2.8 mL of the Coenzyme solution.
   - Mix thoroughly to prepare the Indicator Working Solution.

Indicator Working Solution

- Dilute the sample solution with deionized water according to the following ratios: 1 (undiluted), 1/5, $1/5^2$, $1/5^3$, $1/5^4$, $1/5^5$, and $1/5^6$.

Table S1. Addition Sequence & Amount of Each Solution

	Sample	Blank1	Blank2
Sample solution	20 μL	-	-
Deionized water	-	20 μL	40 μL
Substrate buffer	20 μL	20 μL	20 μL
Enzyme working solution	20 μL	20 μL	20 μL
Indicator working solution	20 μL	200 μL	200 μL

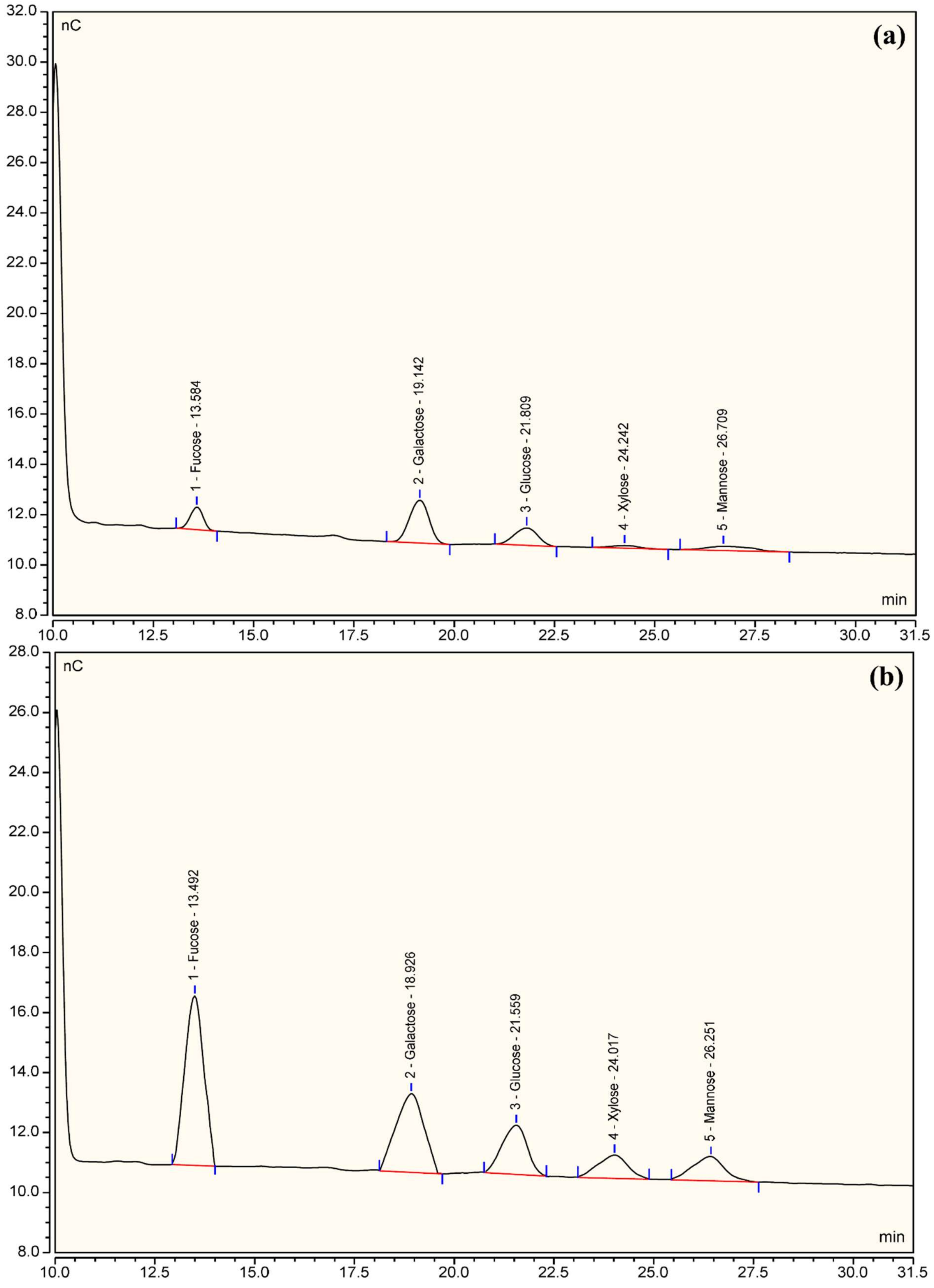

(a)
nC
min
1 - Fucose - 13.584
2 - Galactose - 19.142
3 - Glucose - 21.809
4 - Xylose - 24.242
5 - Mannose - 26.709
(b)
nC
min
1 - Fucose - 13.492
2 - Galactose - 18.926
3 - Glucose - 21.559
4 - Xylose - 24.017
5 - Mannose - 26.251

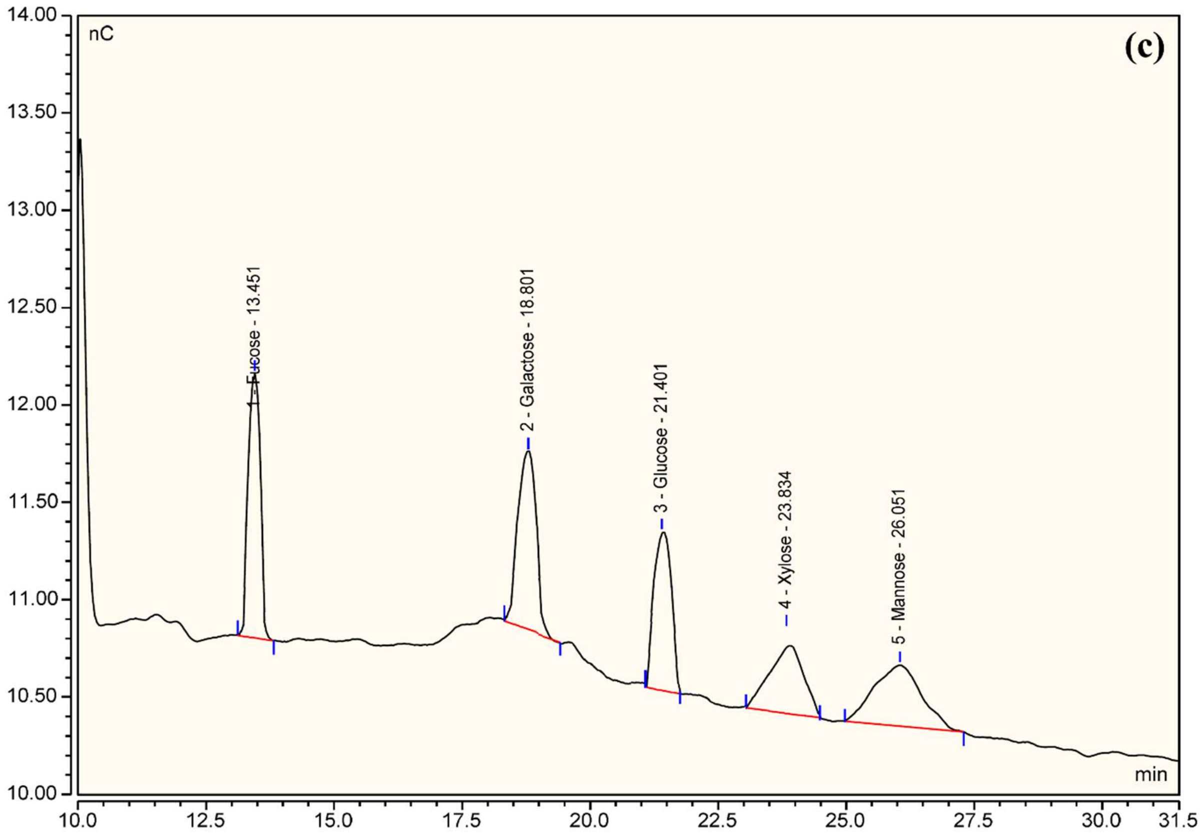

Figure S1. Monosaccharides chromatogram of USEs

(a) USE-B; (b) USE-S; (C) USE-R

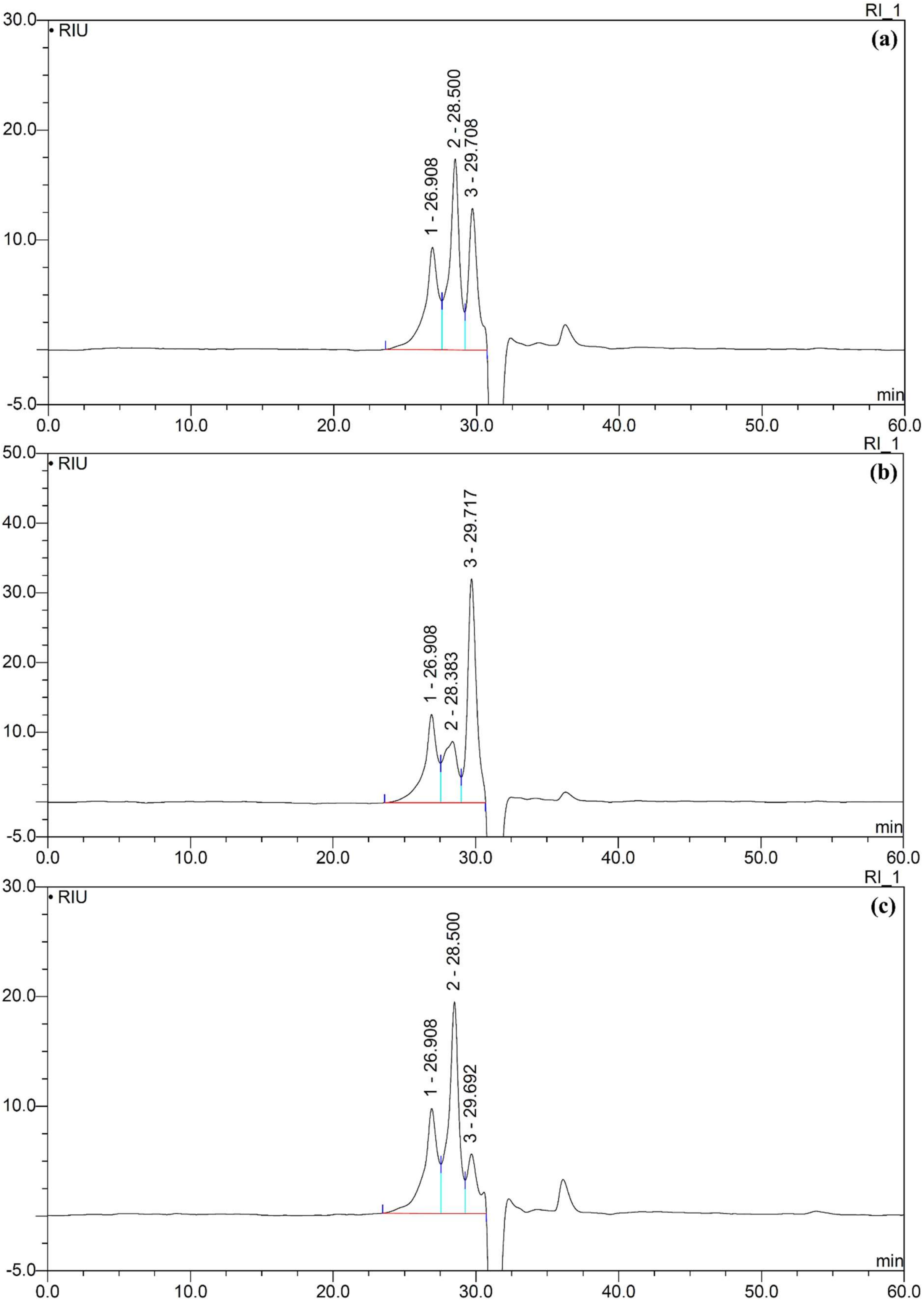

Figure S2. GPC Chromatogram of USEs

(a) USE-B; (b) USE-S; (C) USE-R
